# RISING FROM THE FLOOR IN PERSONS WITH PARKINSON’S DISEASE

**DOI:** 10.1093/geroni/igz038.1768

**Published:** 2019-11-08

**Authors:** Dennis W Klima, Jeremy Stewart, Frank Freijomil, Mary DiBartolo

**Affiliations:** 1 UMES, Princess Anne, Maryland, United States; 2 Salisbury University, Salisbury University, United States

## Abstract

While considerable research has targeted gait, balance and preventing falls in individuals with Parkinson’s disease (PD), less in known about the ability to rise from the floor in this population. The aims of this study were to 1) Examine the relationship between locomotion and physical performance tests and the timed supine to stand performance measure and to 2) Identify both the time required and predominant motor patterns utilized by persons with PD to complete to floor rise transition. A cross-sectional design was utilized. Twenty community-dwelling older adults with PD (mean age 74.8+/-9.5 years; 13 men) performed a standardized floor rise test and locomotion tests in a structured task circuit. Subject demographic and anthropometric data were also collected. Statistical analyses included descriptive statistics and Pearson Product Moment correlations. Fifteen subjects (75%) demonstrated the crouch kneel pattern and fourteen (70%) used an all-4’s strategy to rise to stand. The mean time to rise from the floor was 14.9 (+/- 7.6) seconds and slower than published norms for persons without PD. Nine subjects required the use of a chair to perform floor recovery. Supine to stand performance time was significantly correlated with the: Dynamic Gait Index (r= - 0.66; p<0.002), Five Times Sit to Stand Test (r=0.78; p<0.001), Timed Up and Go Test (r=0.74; p<0.001), and gait velocity (r= -0.77; p<0.001). Rising from the floor demonstrates concurrent validity with locomotion and physical performance tests. Floor recovery techniques can be incorporated in fall prevention initiatives in conjunction with PD symptom management.

